# Insights into human kidney function from the study of *Drosophila*

**DOI:** 10.1007/s00467-023-05996-w

**Published:** 2023-05-12

**Authors:** Sybille Koehler, Tobias B. Huber

**Affiliations:** 1 III. Department of Medicine, University Medical Center Hamburg-Eppendorf, Hamburg, Germany; 2 Hamburg Center for Kidney Health (HCKH), University Medical Center Hamburg-Eppendorf, Hamburg, Germany

**Keywords:** Drosophila melanogaster, Nephrocyte, Podocyte, Model organism, Inter-organ communication

## Abstract

Biological and biomedical research using *Drosophila melanogaster* as a model organism has gained recognition through several Nobel prizes within the last 100 years. *Drosophila* exhibits several advantages when compared to other in vivo models such as mice and rats, as its life cycle is very short, animal maintenance is easy and inexpensive and a huge variety of transgenic strains and tools are publicly available. Moreover, more than 70% of human disease-causing genes are highly conserved in the fruit fly. Here, we explain the use of *Drosophila* in nephrology research and describe two kidney tissues, Malpighian tubules and the nephrocytes. The latter are the homologous cells to mammalian glomerular podocytes and helped to provide insights into a variety of signaling pathways due to the high morphological similarities and the conserved molecular make-up between nephrocytes and podocytes. In recent years, nephrocytes have also been used to study inter-organ communication as links between nephrocytes and the heart, the immune system and the muscles have been described. In addition, other tissues such as the eye and the reproductive system can be used to study the functional role of proteins being part of the kidney filtration barrier.

## *Drosophila melanogaster* as a model organism

The use of *Drosophila melanogaster* as a tool in research has a long-standing history, with the first of six Nobel prizes for research in *Drosophila* being awarded almost 100 years ago in 1933 (Table 1).

**Table 1:** Nobel prizes using *Drosophila*

Year	Researcher	Title
1933	Thomas Hunt Morgan	The role played by chromosomes in heredity
1946	Hermann Joseph Muller	The production of mutations by means of X-ray irradiation
1995	Edward B. Lewis Christiane Nüsslein-Volhard Eric F. Wieschaus	The genetic control of early embryonic development
2004	Richard Axel	Odor receptors and the organization of the olfactory system (mainly rodent work)
2011	Jules A. Hoffmann	The activation of innate immunity
2017	Jeffrey C. Hall Michael Rosbash Michael W. Young	Molecular mechanisms controlling the circadian rhythm

**Fig. 1:**
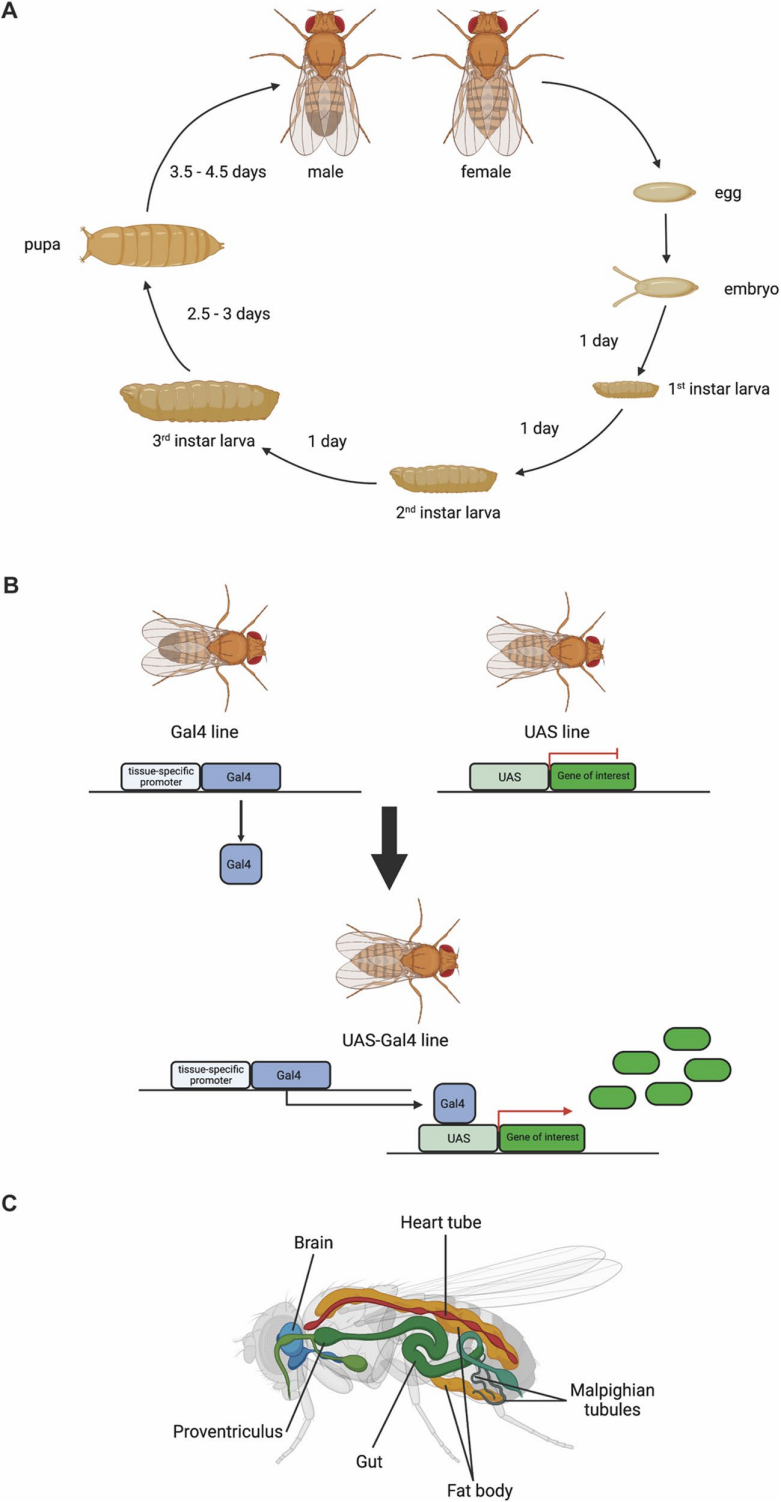
*Drosophila* life cycle and UAS-Gal4 system. **A** The *Drosophila* life cycle is approximately 10 days and can be divided into four stages: embryo, larva, pupa and adults. After mating, female adult flies lay eggs, becoming fertilized embryos. These develop into 1^st^ instar larvae within a day. The 2nd and 3rd instar larval stages are accomplished after one more day each. The development of pupa takes between 2.5 and 3 days. Fully developed adults eclose after 3.5 to 4.5 days.** B** The UAS-Gal4 system is a widely used tool in *Drosophila* research and enables tissue/ cell type specific expression or repression of genes of interest. The Gal4 line contains the tissue-specific promoter followed by the yeast-transcription factor Gal4. The UAS line harbors the Upstream Activator Sequence (UAS) which is located before the DNA sequence of the gene of interest or the respective RNAi for repression purposes. Mating of the two lines will generate flies expressing the Gal4 transcription factor in the intended tissue or cells, which will bind to the UAS sequence enabling expression or repression of the gene of interest. **C** Body plan of *Drosophila melanogaster* illustrating the heart tube, the brain, the gut and the proventriculus as well as the fat body and the Malpighian tubules. Images were created with Biorender.com

These findings helped to make *Drosophila* an important tool in biological and biomedical research. Among the various advantages of the fly model is the short life cycle of *Drosophila*. It takes only 10 days from laying fertilized eggs to adult flies. Within these 10 days flies pass through 4 developmental stages: embryo, larva, pupa, adult (Fig. 1A). The lifetime of *Drosophila* in the lab is between 60 and 80 days, also making it a useful tool in aging research. Moreover, as *Drosophila* can be kept in vials containing sufficient food for several generations, *Drosophila* research is also inexpensive compared to other model organisms such as mice. Further advantages are the easy genetic manipulation using the UAS-GAL4 system, which allows a tissue/cell-type specific expression or depletion of target genes (Fig. 1B), as well as the huge number of transgenic flies available for purchase at organizations such as the Bloomington Drosophila Stock Center (BDSC, Indiana University), the Vienna Drosophila Resource Center (VDRC), the Kyoto Stock Center (DGGR) and the Drosophila Genomics Resource Center (DGRC). Also, the development of novel high-end techniques such as high-resolution imaging, single cell/nucleus RNA sequencing, spatial transcriptomics and proteomic approaches have been successfully used in the fruit fly [1–4].

*Drosophila* is a valuable tool in biomedical research, as more than 70% of human disease-causing genes are conserved in the fly. Moreover, although being an invertebrate, the body organization of the fruit fly is analogous to mammals, making it a useful tool to study the impact of disease-causing genes on organ function and fly physiology (Fig. 1C) [5]. *Drosophila* has an open circulatory system, with only one body fluid, the hemolymph, which is the blood equivalent in insects. The heart tube consists of a single layer of cardiomyocytes and performs pulsatile contractions, which result in hemolymph flow [6]. Given the high conservation of genes and proteins found in *Drosophila* in the mammalian heart, it can be used to investigate pathways and mechanisms influencing heart function in mammals [7, 8]. The liver equivalent is called the fat body and is important for maintaining energy storage, immune response and nutrient sensing [9]. Moreover, *Drosophila* brains are used to investigate complex behaviors such as navigation, learning and courtship, as studies can be performed at the level of single cells, because the fly brain contains only 100,000 neurons compared to around 86 billion neurons in the human brain [10]. The immune system in *Drosophila* is not as complex as in humans, as the major cell types are macrophages, which represent the innate immune system [11]. Equivalents to B and T cells are not known to exist in insects. Macrophages can be resident or floating and phagocytose PAMPs and DAMPs circulating in the hemolymph. Taken together, *Drosophila* is a great tool to study cellular functions and physiological mechanisms, which might be relevant for mammalian physiology as well [5]. In this review, we will describe different *Drosophila* organs, which can be used in nephrology research, known inter-organ communication pathways that involve nephrocytes and ways in which *Drosophila* can be used in translational research.

## The *Drosophila* kidney

The *Drosophila* kidney consists of two different cell types/tissues: the Malpighian tubules and the nephrocytes. Malpighian tubules resemble the tubular apparatus of the mammalian kidney and are formed by an epithelial single layer. The four tubules evolve by growing out of the mind-hind-gut junction. Among the cell types identified in Malpighian tubules are principal and stellate cells [12]. In addition, at the distal end of each tubule, a tip cell was identified, which regulates tissue architecture and position in the body cavity [13]. Malpighian tubules can take up products from the hemolymph and produce primary urine by potent active cation transport [14]. In the proximal part reabsorption takes place similar to reabsorption in the mammalian tubules [15]. The so-called Ramsay assay can be performed to assess urine formation and urine composition. In addition, several studies have been done to identify and characterize pattern formation in tubules along the distal–proximal axis in Malpighian tubules [12, 16–19].

**Fig. 2:**
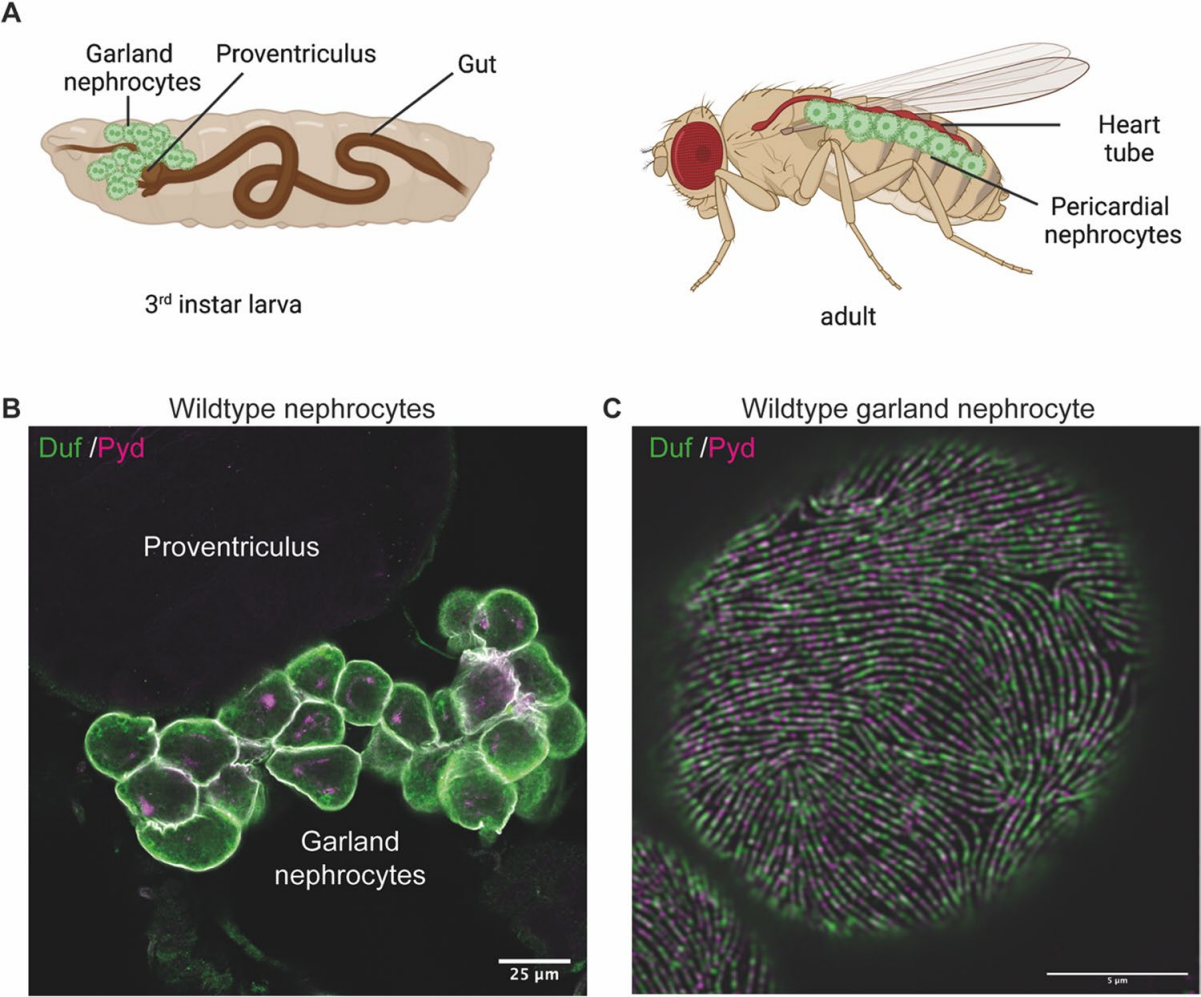
*Drosophila* nephrocytes. **A** In *Drosophila* there are two different populations of nephrocytes: garland nephrocytes, which are located around the esophagus, and pericardial nephrocytes, which are positioned along the heart tube.** B** Visualization of the nephrocyte diaphragm proteins Duf (NEPH) and Pyd (ZO-1) show the arrangement of garland cells around the esophagus and on top of the proventriculus. Scale bar = 25 μm.** C** High-resolution microscopy visualizing Duf and Pyd reveals a finger-print like pattern of the nephrocyte diaphragm in wildtype garland nephrocytes. Scale bar = 5 μm

The second kidney cell type, the nephrocyte, occurs in two different populations; the garland nephrocytes and the pericardial cells. Garland nephrocytes are grouped as a necklace around the esophagus and lie on top of the proventriculus, while pericardial cells are positioned along the heart tube (Fig. 2A, B). One major functional role of nephrocytes is the filtration of hemolymph, including uptake, endocytosis and processing of toxins and waste products [20]. Performing a single nucleus RNA sequencing approach enabled the generation of a fly kidney atlas, in which the different segments of Malpighian tubules and the two nephrocyte populations could be distinguished based on gene expression patterns [14]. From earlier studies it was known that nephrocytes express cubillin and amnionless, two proteins which are found in mammalian tubules [21, 22]. Based on these findings nephrocytes are described as exhibiting similarities to both podocytes and proximal tubules [22]. However, in the recent fly kidney atlas the different *Drosophila* cell types were compared with available mammalian kidney single cell data and revealed that adult garland and pericardial nephrocytes share the same gene signature with podocytes and present with a partial overlap with PECs [14]. Moreover, the previously described slit diaphragm homologs such as sticks-and-stones (Sns; Nephrin homolog) and dumbfounded (Duf/kirre; NEPH homolog) were also identified in both nephrocyte populations. Nonetheless, the endocytic function similar to proximal tubular cells is also evident, hence a functional similarity to this kidney compartment offers additional avenues to model not only podocyte function, but also aspects of (proximal) tubular functions.

In addition to the podocyte-specific gene signature, nephrocytes share a highly similar morphology with mammalian podocytes. They form membrane invaginations, which result in a lacunae network. The lacunae are flanked by foot processes, which are attached to a basement membrane and between the foot processes the nephrocyte diaphragm is formed (Fig. 2C). This specialized cell-contact is built out of Sns and Duf, the homologous proteins to Nephrin and NEPH [20, 23]. Moreover, this contact is size- and charge-selective and filtered molecules will then end up in the lacunae system, where they will be endocytosed [20]. Of note, although nephrocytes and podocytes are highly similar in their molecular make-up and morphology, nephrocytes are not in contact with endothelial cells, as these are absent in *Drosophila.* Moreover, the nephrocyte diaphragm is formed between foot processes originating from only one cell. However, *Drosophila* nephrocytes proved to be an ideal tool to investigate mechanisms of podocyte injury in greater detail.

One major difficulty in podocyte research is the accessibility of podocytes, as isolation of these cells can only be done via labeling and fluorescence activated cell sorting (FACS), which involves mechanical stress and enzymatic digestion. Therefore, one can easily envision that podocytes do not represent the in vivo situation after running through the isolation protocol. The isolation of garland nephrocytes and pericardial cells is very easy and fast and does not involve mechanical stress or enzymatic digestion. Due to their high similarity and easy accessibility several studies have utilized *Drosophila* nephrocytes to study signaling pathways and specific patient mutations identified in glomerular disease. These pathways involve the actin-cytoskeleton, cell polarity signaling, mitochondria associated signaling and the endocytic machinery, among others (for details see Table 2).

**Table 2:** Selected genes and pathways studied in *Drosophila* nephrocytes.

Gene (*Drosophila*)	Putative human ortholog(s)	Pathway/ Compartment	Reference
*Sticks-and-Stones*	NPHS1*	Nephrocyte diaphragm	[20, 23]
*Dumbfounded/Kirre*	KIRREL1* *KIRREL2* *KIRREL3*	Nephrocyte diaphragm	[20, 23, 26]
*Mec2*	NPHS2*	Nephrocyte diaphragm	[20]
*Cindr*	*SH3KBP1* *CD2AP*^+^	Nephrocyte diaphragm	[20, 27]
*Polychaetoid*	*TJP1* *TJP2* *TJP3*	Nephrocyte diaphragm	[20, 28]
*Src64B*	*FYN* *SRC* *YES1*	Nephrocyte diaphragm	[29]
*Knot*	*EBF1* *EBF2* *EBF3* *EBF4*	Transcription factor	[30]
*dKlf15*	*KLF15*	Transcription factor	[31]
*CG32105*	*LMX1A* *LMX1B*^+^	Transcription factor	[27]
*Nup93*	NUP93*	Nuclear pore complex	[32]
*Nup160*	NUP160*	Nuclear pore complex	[33]
*Moesin*	*EZR* *RDX* *MSN*	Actin-cytoskeleton	[34]
*Actn*	*ACTN1* *ACTN2* *ACTN3* *ACTN4*^+^	Actin-cytoskeleton	[27, 35, 36]
*Arhgap92B*	*ARHGAP24*^*(*+*)*^	Actin-cytoskeleton	[27, 35, 37]
*Zipper*	*MYH9*^*(*+*)*^ *MYH10* *MYH11* *MYH14*	Actin-cytoskeleton	[27, 35, 38]
*Scraps*	*ANLN*^+^	Actin-cytoskeleton	[35]
*Kank*	*KANK1* KANK2* *KANK4*	Actin-cytoskeleton	[27, 35]
*RhoGDI*	ARHGDIA* *ARHGDIB* *ARHGDIG*	Actin-cytoskeleton	[27, 35, 39]
*Myo61F*	*MYO1C* *MYO1E*^+^ *MYO1H*	Actin-cytoskeleton	[35]
*Formin3*	*INF2*^+^ *FHDC1*	Actin-cytoskeleton	[35, 40]
*Tiggrin*		Actin-cytoskeleton	[39]
*Titin*		Actin-cytoskeleton	[39]
*Coracle*	*EPB41* *EPB41L1* *EPB41L2* *EPB41L3*	Actin-cytoskeleton	[39]
*Tropomyosin2*	*TPM1* *TPM2* *TPM3* *TPM4*	Actin-cytoskeleton	[39]
*CG1674*	*SYNPO*	Actin-cytoskeleton	[27, 39]
*Lasp*	*LASP1*	Actin-cytoskeleton	[41]
*Multiple edematous wings*	ITGA3* *ITGA6* *ITGA7*	ECM-interaction	[27, 35]
*Myospheroid*	*ITGB1*^*(*+*)*^ *ITGB2* *ITGB3* *ITGB4* *ITGB7*	ECM-interaction	[27, 35, 42]
*Vinculin*	*VCL*	ECM-interaction	[39]
*Viking*	*COL4A1*^*#*^	ECM-interaction	[35]
*Crumbs*	*CRB1* *CRB2*^+^	Cell polarity signaling	[34]
*Stardust*	*PALS1*	Cell polarity signaling	[34, 43]
*Patj*	*PATJ* *MPDZ*	Cell polarity signaling	[34, 43]
*Bazooka*	*PARD3* *PARD3B*	Cell polarity signaling	[39, 43]
*aPKC*	*PRKCI* *PRKCZ*	Cell polarity signaling	[39, 43]
*Par6*	*PARD6A* *PARD6B PARD6G*	Cell polarity signaling	[39, 43]
*Discs large 1*	*DLG1* *DLG2* *DLG3* *DLG4*	Cell polarity signaling	[43, 44]
*Scribble*	*SCRIB*	Cell polarity signaling	[43, 44]
*Lethal (2) giant larvae*	*LLGL1* *LLGL2*	Cell polarity signaling	[43, 44]
*Par-1*	*MARK1* *MARK2* *MARK3* *MARK4*	Cell polarity signaling	[43, 44]
*Lkb1 kinase*	*STK11*	Cell polarity signaling	[43, 44]
*Vps34*	*PIK3C3*	Endocytosis	[45]
*Rabphilin*	*RPH3A* *DOC2A* *DOC2B*	Endocytosis	[46]
*Shibire*	*DNM1* *DNM2* *DNM3*	Endocytosis	[25, 47]
*Cubilin*	*CUBN*^*(*+*)*^	Endocytosis	[22, 48, 49]
*Cubilin2*	*CUBN*^*(*+*)*^	Endocytosis	[48, 49]
*Amnionless*	*AMN*	Endocytosis	[22, 48]
*Rab5*	*RAB5A* *RAB5B* *RAB5C*	Endocytosis	[25, 50]
*Rab7*	*RAB7A*	Endocytosis	[25, 50]
*Rab11*	*RAB11A* *RAB11B*	Endocytosis	[25, 50]
*Rab39*	*RAB39A* *RAB39B*	Endocytosis	[51]
*Flotillin2*	*FLOT2*	Endocytosis	[25]
*Arf79F*	*ARF1* *ARF3*	Endocytosis	[47]
*Clathrin heavy chain*	*CLTC* *CLTCL1*	Endocytosis	[47]
*Clathrin light chain*	*CLTA* *CLTB*	Endocytosis	[47]
*Lap*	*SNAP91* *PICALM*	Endocytosis	[47]
*Auxilin*	*GAK* *DNAJC6*	Endocytosis	[47]
*Hsc70-4*	*HSPA1B* *HSPA2* *HSPA8*	Endocytosis	[47]
*Gapvd1*	*GAPVD1*^(^*^)^	Endocytosis	[52]
*Sec20*	*BNIP1*	Endocytosis	[53]
*Coq2*	*COQ2*^*§*^	Mitochondria	[27, 35]
*Pdss2*	*PDSS2*^*§*^	Mitochondria	[27, 35]
*Coq6*	*COQ6*^*§*^	Mitochondria	[27]
*Mechanistic Target of rapamycin*	*MTOR*	TOR signaling/autophagy	[54, 55]
*Tsc1*	*TSC1*	TOR signaling/autophagy	[54]
*Autophagy-related 5*	*ATG5*	TOR signaling/autophagy	[54]
*Hippo*	*STK3* *STK4*	HIPPO signaling	[55]
*Yorkie*	*YAP1* *WWTR1*	HIPPO signaling	[55]
*Shaggy*	*GSK3A* *GSK3B*	WNT signaling	[56]
*Armadillo*	*CTNNB1* *JUP*	WNT signaling	[55]
*Pangolin*	*TCF7L2* *LEF1*	WNT signaling	[55]
*Dab*	*DAB1* *DAB2*	Notch signaling	[57]
*Cheerio*	*FLNA* *FLNB* *FLNC*	Mechanotransduction	[24, 55]
*Piezo*	*PIEZO1* *PIEZO2*	Mechanotransduction	[58]
*Skittles*	*PIP5K1A* *PIP5K1B* *PIP5K1C*	Phospholipid pathway	[59]
*PTEN*	*PTEN*	Phospholipid pathway	[59]
*Sply*	SGPL1*	Lipid metabolism	[60]
*APOL1*	*APOL1*^+^	Lipid metabolism	[61–63]
*Midway*	*DGAT1*	Lipid metabolism	[64]
*Hnf4*	*HNF4A^* *HNF4G*	Lipid metabolism	[65]
*Exo70*	*EXOC7*	Exocyst complex	[59]
*Tbc1d8-9*	*TBC1D8* TBC1D8B* *TBC1D9* *TBC1D9B*	GTPase-activating protein	[66, 67]
*Rho1*	*RHOA* *RHOB* *RHOC*	GTPase	[39, 58, 68]
*Rac1*	*RAC1* *RAC2* *RAC3*	GTPase	[39, 58, 68]
*Cdc42*	*CDC42*	GTPase	[39, 58, 68]
*Rap1*	*RAP1A* *RAP1B* *RAP1BL*	GTPase	[69]
*c3g*	*RAPGEF1*	GTPase	[70]
*Tcs3*	OSGEP*	KEOPS-complex	[71]
*Stim*	*STIM1* *STIM2*	Ca^++^-signaling	[72]
*Orai*	*ORAI1* *ORAI2* *ORAI3*	Ca^++^-signaling	[72]

To identify human orthologs the “Alliance of Genome Resources” and “MARRVEL” databases have been used. All orthologs with a DIPOT-score of at least 50% are listed in the table. Disease association has been verified using the database ‘Online Mendelian Inheritance in Man’ (OMIM). () indicate that disease association is not mentioned on OMIM, but has been published elsewhere (publication is mentioned in the table). The table lists only selected disease caused by mutations in the respective genes. Differential regulation upon glomerular injury is not included here. *: nephrotic syndrome, ^+^: FSGS, ^#^: Alport syndrome, ^§^: Coenzyme Q10 deficiency, and ^: Fanconi renotubular syndrome with maturity-onset diabetes of the young

In recent years, many technical advances have also been applied to nephrocytes. High-resolution microscopy to visualize the nephrocyte diaphragm as well as omics approaches to study gene and protein signatures have been implemented successfully [14, 24]. In a recent publication, two novel fly strains were described, which are useful tools to study Sns (Nephrin) trafficking and enable studies to investigate nephrocyte diaphragm maintenance and dynamics. These fly strains were generated by introducing either a GFP- or a Myc-tag into the endogenous Sns locus [25].

## The *Drosophila* eye and reproductive system in nephrology research

Duf, the NEPH homologue and Sns, the Nephrin homologue are not exclusively expressed in nephrocytes, but are also present in the *Drosophila* eye, testis, and ovaries. Hence, these organs can also be used to assess molecular function, in particular of different isoforms and mutated variants of these proteins.

The *Drosophila* eye is a compound eye and consists of a lens, retina and pigment layer, which are highly compressed [73]. The eye contains around 700 ommatidia, consisting of eight photoreceptor neurons, four lens-secreting cone cells and two primary pigment cells [73, 74]. During late larval stages a wave of differentiation generates loosely arranged ommatidia and in young pupa undifferentiated interommatidial precursor cells develop into secondary and tertiary pigment cells, forming a honeycomb-like pattern [74, 75]. The secondary and tertiary pigment cells serve as an insulating lattice, thereby preventing light from passing between ommatidia. This highly specialized pattern evolves from a precisely organized developmental process involving coordinated cell signaling, cell proliferation, cell death and cell movements. This process is not only of highest interest for developmental biologists, as pathways such as EGFR/Ras, Notch, Dpp (BMP), Wg (Wnt), and Hedgehog are involved during development, but can also be used to understand cell–cell contact formation involving classical junction proteins such as cadherins [76] as well as the adhesion-like transmembrane proteins Hibris (Hbs) and Roughest (Rst), which belong to the Nephrin superfamily and are orthologues of Nephrin and NEPH [73, 77]. Loss of either of these proteins results in an incorrect patterning of the interommatidial precursors and a rough eye phenotype [74]. Interestingly, Sns and Duf, which are the homologues of Nephrin and NEPH, respectively, have redundant functions in the eye development [26, 74].

In addition to the expression and functional role of Duf and Sns in *Drosophila* eyes, they have also been described in the reproductive system of flies. Both Duf and Sns are expressed in myoblasts developing into testis muscle cells [78]. Although they play an important role in myoblast fusion, loss of either of the proteins did not impact male fertility [78]. Additional studies revealed expression of Duf in pupa and adult ovaries, where it localizes to the nurse cell membranes and the ring canals, but loss of Duf did not impact female fertility [79]. Future studies need to be done to further delineate the functional role of Duf and Sns in the cells of the reproductive systems of flies.

## Nephrocytes and their role in inter-organ communication

Interestingly, previous studies showed a link between podocytes and other organs and tissues such as the heart, immune system and muscles during disease states [80–82]. Hence, it is very likely that podocytes are involved in inter-organ communication. *Drosophila* represents the ideal tool to further investigate this hypothesis and whether nephrocytes monitor physiological states during health and disease and modulate the function of other organs accordingly to respond to perturbations. Due to their unique position (along the heart tube and around the esophagus (Fig. 2) nephrocytes are exposed to virtually all systemic changes and influences in the fly body, such as changes of hemolymph composition (ions, nutients, and pathogens) and physical properties such as circulation and pressure. This concept can be translated into the mammalian organism, as all organs are supplied by blood and are dependent on its physiological composition and physical properties. In glomerular disease, which often results in chronic kidney disease (CKD), podocyte injury is a common hallmark. Podocytes are post-mitotic cells and undergo morphological rearrangement processes during injury, followed by detachment from the basement membrane. Thus, capillaries remain blank and proteinuria occurs, causing changes in blood composition and circulation.

CKDs are often accompanied by other disorders such as cardiac diseases. As early as 1840, Bright had already described a changed heart morphology in patients suffering from kidney diseases [83]. Since then several researchers and studies have investigated the so-called cardio-renal syndrome, in which acute or chronic dysfunction of one organ induces acute or chronic dysfunction in the other organ [84]. The pathophysiology of the cardio-renal syndrome includes changing blood pressure, which results in changing blood flow in the glomerular capillaries [84], and the release of inflammatory mediators after acute kidney injury, resulting in cardiac injury [80]. Moreover, it was previously shown that cardio-renal syndrome also causes glomerular injury and podocyte loss [85], but molecular mechanisms of podocyte injury and whether podocytes might influence the heart function remain elusive.

As genes and proteins are highly similar between the mammalian and the *Drosophila* heart, it is a widely-used and useful tool to study mechanisms in heart function. Hence, the cross-talk between heart and nephrocytes has been studied in the fruit fly. In detail, it was shown that nephrocytes (pericardial cells), which are localized along the heart tube in *Drosophila*, produce extracellular matrix (ECM) components during embryogenesis and are important to maintain normal heart function [86]. Interestingly, the absence of nephrocytes (induced by dKlf15 depletion) caused a severe cardiomyopathy phenotype, which is characterized by lengthening of the diastolic interval [86]. This cardiomyopathy phenotype is a result of elevated Secreted Protein Acidic and Rich in Cysteine (SPARC) levels, a matricellular protein, which is involved in mammalian cardiac function, in the absence of nephrocytes [86].

Although initial studies confirmed the inter-organ communication hypothesis and the involvement of podocytes [81, 82, 84], several underlying mechanisms and communication ways are still not known. In a recent study, Solagna et al. discovered a novel inter-organ signalling mechanism linking skeletal muscle wasting with CKD [82]. During CKD kidney fibroblasts and cells of the juxtaglomerular apparatus produce and secrete pro-cachectic factors, among them Activin A, resulting in elevated blood levels of these factors and subsequent skeletal muscle wasting [82]. A similar mechanism was observed by Mulderrig et al., as the production of endogenous formaldehyde induced transcriptional stress in nephrons resulting in an endocrine weight loss response [87].

These studies show an involvement of the kidney in inter-organ communication, but the role of podocytes in this cellular cross-talk is mainly unknown. In recent years studies utilizing nephrocytes tackled this question and showed a link between nephrocytes and the gut, immune system and muscle/neuronal tissue [21, 88]. In the work of Feng et al. it was shown that nephrocyte-mediated reabsorption of proteins from the hemolymph modulates the fly’s lifespan by regulating proteostasis in muscle and brain tissue [21]. Interestingly, the absence of nephrocytes (induced by depletion of dKlf15) resulted in an increased resistance to infection and a shortened lifespan in *Drosophila* [88]. This finding is explained by an uptake of microbiota-derived PGN (peptidoglycan) by nephrocytes, which prevents Toll pathway activation thereby contributing to immune homeostasis [88]. These studies confirm that nephrocytes are sampling the hemolymph constantly thereby modulating fly physiology. Whether nephrocytes also secrete factors to influence other organs and tissues such as the heart and macrophages upon alterations in hemolymph composition remains unknown.

## Clinical implications of *Drosophila* research

*Drosophila* is an effective tool to study the functional role of human disease genes in a huge variety of disease entities. The most prominent ones are neurological disorders such as Alzheimer’s and Parkinson’s disease, cancer, and dysmorphologies, which arise due to mutations in genes essential for development. In addition, cardiovascular and kidney as well as metabolic and storage diseases and immunological disorders are studied in flies [89]. Interestingly, although *Drosophila* belongs to the invertebrates, more than 70% of human-disease causing genes are conserved [89]. Another advantage of *Drosophila* as a model organism for human diseases is the lower redundancy when compared to mammalian model organisms or cell culture systems (also depicted in Table 2) [90]. This lower redundancy, together with a high conservation of genetic pathways and protein–protein interactions, which are controlled by a disease-associated gene, makes it easier to characterize gene functions in disease. Also, *Drosophila* can be used for so-called second-site modifier screens. In detail, these genetic screens are performed to identify mutations, which are recessive in wildtype flies, but become dominant in mutant flies. Thus, these mutations will enhance or suppress the starting phenotype of the mutant flies, making them modifiers. This approach has been used very successfully in the identification of novel human tumor-suppressor genes [89].

With an increase of genome and exome sequencing of patient material, novel patient mutations are identified, but the functional role of these mutated genes remains mostly unknown. To overcome this limitation, clinicians and model organism researchers have teamed up to develop valuable tools and networks with the goal to understand the functional role of disease-causing genes. Among these tools are data-bases such as MARRVEL (Model organism Aggregated Resources for Rare Variant Exploration), UDN (Undiagnosed Disease Network) and RDMM (Rare Diseases Models and Mechanisms) [90], which provide useful tools to identify orthologues to human genes as well as additional information such as disease-association, expression, sequencing, and protein data. Moreover, MARRVEL also provides data about available drugs via a link to the PHAROS database. Once novel patient mutations have been identified, it is easy to generate flies expressing the human variant and study its functional role in the organ or tissue of interest.

**Fig. 3:**
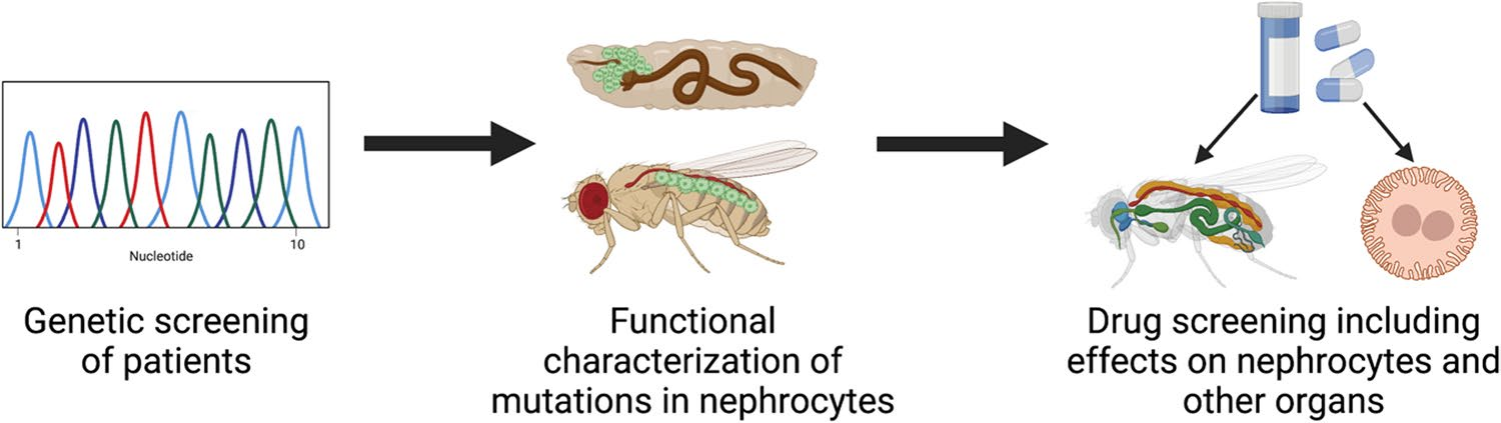
Translational pipeline. Novel patient mutations causing podocyte injury can be identified by genetic screening. The functional role of these mutations can then be assessed in *Drosophila* nephrocytes, and applying omics approaches and utilizing sensor fly strains can reveal the involvement of downstream signaling pathways. Potential novel drugs can then be tested in regard to their effect on nephrocytes expressing patient mutations and other organs as well, to investigate the effect on the whole organism. Images created with BioRender.com

Within this review we focus on nephrocytes as a model for kidney disease. Hence, in the next part, we will summarize how nephrocytes have been used in translational nephrology and how they could be used in the future. As outlined in Table 2, several genes have been studied in *Drosophila* nephrocytes, which play a role in human kidney disease. Often these genes are identified in the clinical screening of patients and are then tested for their functional role in the nephrocyte model. By doing so, identified patient mutations can be functionally characterized, which not only involves assessment of nephrocyte biology but also the identification of regulated downstream signaling pathways and targets. Also, genetically engineered flies expressing the mutated human variants can be used for drug screening purposes. Potential novel drugs can be applied by incubating isolated nephrocytes or by oral delivery via the food. This will enable assessment of effects on nephrocyte biology, but also more generally, on other organs as well. In addition, downstream signaling pathways, which are altered due to expression of the mutated patient variant can be investigated using available sensor fly strains in the absence or presence of the above-mentioned drugs. Thus, a pipeline (Fig. 3) can be implemented in which (1) novel mutations are functionally characterized in nephrocytes; (2) altered downstream signaling pathways and targets are identified; (3) potential novel drugs are tested in regard to their effect on nephrocytes and other organs; and can help determine (4) whether these drugs impact on altered downstream signaling pathways and targets. Although *Drosophila* is an invertebrate, the results obtained within such a translational pipeline are beneficial in comparison to in vitro systems, as general effects on the whole organism can be assessed as well. The most promising drugs can then be tested in more expensive and time-consuming mouse models or human organoids, prior to clinical phase studies.

In addition to this proposed translational pipeline, human disease associated with kidney malfunction can be mimicked in the fly model. One very well described model is diabetic nephropathy. Cagan and colleagues established a model in which feeding chronic high dietary sucrose causes a nephrocyte phenotype phenocopying human diabetic nephropathy [30]. Moreover, using their fly model they identified an OGT-Polycomb-Knot-Sns pathway, which mediates nephrocyte dysfunction upon high dietary sugar. Based on their findings, they studied expression levels of the Knot orthologue EBF2 in diabetic nephropathy patient derived glomerular tissue and diabetic mouse models and could confirm an increase of the transcription factor upon disease.

Another example, where *Drosophila* has helped to gain some insights into human disease, is apolipoprotein-L1 (APOL1) associated kidney disease. Risk variants (G1 and G2) have been identified and described to cause cell injury also in podocytes, but underlying mechanisms are only poorly understood. To study the functional role of the wildtype and the risk variants and how they contribute to the disease phenotype, flies expressing the three different variants have been generated and analyzed [61–63]. In all three studies, expression of the risk variants in nephrocytes resulted in enhanced endocytic function. Interestingly, nephrocytes expressing the risk variants are lost during aging and presented with a hypertrophy phenotype [61, 62]. In addition, the expression of the APOL1 risk variants has been linked to ER stress in nephrocytes and wing discs recently [63]. Of particular interest is the reduced APOL1-mediated cell death after pharmacological inhibition, suggesting ER stress as a central pathway in the pathogenesis of APOL1-associated nephropathies [63].

Taken together, the model organism *Drosophila melanogaster* is not only a very useful tool to characterize protein function and pathways in different organs, but can also be used in translational nephrology research in the future.
